# The Live-Attenuated PruΔ*gra47* Strain of *Toxoplasma gondii* Confers Protective Immunity Against Acute and Chronic Toxoplasmosis in Mice

**DOI:** 10.3390/ani16131964

**Published:** 2026-06-25

**Authors:** Chen-Ran Tian, Xing Tian, Shu-Min Zhao, Zhi Zheng, Wen-Bo Hao, Xing-Quan Zhu, Xiao-Nan Zheng

**Affiliations:** Shanxi Key Laboratory of Animal Disease Research, Prevention and Control, College of Veterinary Medicine, Shanxi Agricultural University, Jinzhong 030801, China; chenrantian2019@163.com (C.-R.T.); txing2001@163.com (X.T.); 18647322134@163.com (S.-M.Z.); 15863201790@163.com (Z.Z.); 16696739873@163.com (W.-B.H.)

**Keywords:** *Toxoplasma gondii*, PruΔ*gra47*, live-attenuated vaccine, acute and chronic infection, immune responses

## Abstract

*Toxoplasma gondii* is an apicomplexan parasite that causes toxoplasmosis, a widespread zoonotic disease leading to serious public health concerns and economic losses to animal husbandry. Currently, highly effective vaccines against toxoplasmosis remain unavailable. This study aimed to investigate the safety, immunogenicity, and protective efficacy of the *gra47*-deficient mutant strain PruΔ*gra47* as a live-attenuated vaccine candidate. We evaluated the virulence of PruΔ*gra47* in a mouse model, determined the optimal immunization dose, and measured serum antibody levels and cytokine profiles. Then, mice immunized with PruΔ*gra47* were challenged with different *T. gondii* strains to assess protection against acute and chronic infection. PruΔ*gra47* displayed significantly attenuated virulence and its ability to form cysts was weakened. Vaccination with 5 × 10^6^ tachyzoites elicited predominantly Th1-skewed immune responses. Immunization with PruΔ*gra47* provided complete protection against challenge infection with relatively low-virulent PYS strain and homologous Pru strain, prolonged survival against the highly virulent RH strain, and achieved a 90% survival rate with reduced brain cyst burden under chronic challenge. In conclusion, PruΔ*gra47* is relatively safe and immunogenic in the murine model, and is worth being evaluated in food-producing animals and cats.

## Simple Summary

Toxoplasmosis is a common zoonotic disease caused by the apicomplexan parasite *Toxoplasma gondii*, which can infect most warm-blooded animals and cause illness in humans, especially those with weakened immune systems. Currently, no safe and highly effective vaccine is widely available for preventing this parasitic zoonosis, highlighting an urgent need for new preventive strategies. In this study, we evaluated a genetically modified parasite strain PruΔ*gra47* as a potential live-attenuated vaccine against toxoplasmosis in a murine model. We found that the virulence of this mutant strain was significantly attenuated and its ability to form cysts in mouse brains was weakened. Vaccination with PruΔ*gra47* effectively stimulated strong protective immune responses in mice. Immunization with PruΔ*gra47* provided complete protection against mild infection with low-virulent parasite strains, prolonged survival following challenge with highly virulent strains, and reduced cyst formation during chronic infection. These findings indicate that PruΔ*gra47* is attenuated and immunogenic in the mouse model. Further studies of the protective immunity induced by vaccination with PruΔ*gra47* in food-producing animals and cats against toxoplasmosis are warranted.

## 1. Introduction

*Toxoplasma gondii* is an obligate intracellular protozoan that causes toxoplasmosis, one of the most widespread zoonotic diseases worldwide, capable of infecting virtually all warm-blooded animals, including humans [1]. Transmission occurs via ingestion of undercooked meat containing tissue cysts, consumption of food, vegetables or water contaminated with sporulated oocysts shed by infected felids, or congenitally from mother to fetus [2].

In immunocompetent individuals, *T. gondii* infection generally manifests as asymptomatic. However, in immunocompromised patients (e.g., patients with HIV/AIDS, organ transplant recipients) and pregnant women, primary infection or reactivation of latent infection can lead to severe, life-threatening diseases [3]. The most common manifestation is *Toxoplasma* encephalitis, which presents as multiple discrete brain lesions and, if untreated, is universally fatal [4]. In pregnant women, acute infection can result in fetal malformations, miscarriage or congenital toxoplasmosis, which commonly manifests as chorioretinitis and neurological complications in affected fetuses and newborns [1,5]. Additionally, toxoplasmosis imposes a significant economic burden on livestock production, as infected animals may experience reproductive disorders, including abortion and reduced fertility [6]. Therefore, effective prevention and treatment strategies are urgently needed.

Currently, the most effective clinical treatment for toxoplasmosis is the combination of sulfadiazine and pyrimethamine [7]. Although this treatment effectively suppresses the proliferation of tachyzoites, it is associated with significant toxicity and side effects. Moreover, the regimen does not eliminate bradyzoites residing within tissue cysts, which persist for the lifetime of the host and can reactivate upon immunosuppression [7]. No currently approved drug is active against the latent cyst stage. Consequently, the development of a vaccine that can induce long-term protection and prevent the formation of tissue cysts remains the most promising strategy for toxoplasmosis control.

Considerable progress has been made in developing *Toxoplasma* vaccines, including inactivated, subunit, DNA, protein, and live-attenuated vaccines [8,9]. Live *T. gondii*-based vaccines trigger strong cellular and humoral immunity and provide effective protection against heterologous pathogen infection [10]. The primary approach to generate live-attenuated vaccines currently involves targeted genetic disruption to reduce virulence. However, existing live-attenuated candidates still face challenges, including the risk of virulence reversion and illness caused by inadequate attenuation [11].

The only commercially approved toxoplasmosis vaccine is Toxovax^®^ (S48 strain), which was generated by more than 3000 serial passages in mice and lost its ability to form tissue cysts. It is used in sheep to prevent congenital toxoplasmosis and reduce parasite load in meat [12]. Nevertheless, its short shelf life, and inability to completely eliminate the parasite limit its widespread use [13].

The widespread development and application of genetic engineering technologies have enabled the generation of numerous live-attenuated *Toxoplasma* vaccine candidates by disrupting genes related to virulence and metabolism. For example, RHΔ*tkl1* induces robust humoral and cellular immune responses that effectively combat *Toxoplasma* infection [14]; ME49Δ*cdpk3* significantly reduces cyst formation and provides protection against acute and chronic *Toxoplasma* infections [15]. PruΔ*gra72* significantly attenuates parasite virulence and induces Th1-biased protective immunity against both acute and chronic *T. gondii* infections in mice [16]. PruΔ*pp2a-c* abolishes cyst formation, elicits strong humoral and cellular immune responses, and confers protection against diverse *T. gondii* strains in mice and cats [17]. These studies demonstrate the feasibility of gene-edited live-attenuated vaccines.

Dense granule proteins (GRAs) are key secretory proteins of *T. gondii* that play important roles in parasite virulence and host immune modulation [18]. Several GRAs have been characterized as essential for the integrity of the parasitophorous vacuole membrane (PVM), such as GRA17 and GRA23 [19]. Recently, we found that genetic deletion of *gra47* resulted in decreased PVM permeability, impaired parasite proliferation, and markedly reduced virulence [20]. GRA47 is proposed to interact with GRA72 and may be involved in maintaining PVM permeability [21]. Importantly, mice infected with the PruΔ*gra47* strain survived and exhibited no overt clinical signs, suggesting that this strain is relatively safe and attenuated.

To date, it remains unknown whether the PruΔ*gra47* strain can serve as a live-attenuated vaccine to confer protection against acute and chronic toxoplasmosis. In this study, we evaluated the immunoprotective potential of PruΔ*gra47* in a mouse model. Specifically, we assessed its ability to protect against lethal acute infection and to reduce brain cyst burden following chronic challenge, and we characterized the humoral and cellular immune responses it elicits. We aimed to determine whether PruΔ*gra47* represents a safe, effective, and practical vaccine candidate for the prevention of toxoplasmosis.

## 2. Materials and Methods

### 2.1. Mice and Parasites

This study used eight-week-old female Kunming mice (Beijing Sibeifu Biotechnology Co., Ltd.; Beijing, China) which are an outbred strain widely used in preclinical vaccine evaluations [9,22]. All mice were housed under specific pathogen-free (SPF) conditions at 25 °C with 50–60% relative humidity and had *ad libitum* access to clean food and water. The mice were acclimated for one week prior to the start of the experiments, and were randomly assigned to different groups by the method of blind grabbing without any specific selection criteria to ensure unbiased distribution of animals across experimental groups. Animal experiments were conducted in accordance with the principles of minimizing animal distress and protecting animal welfare.

The following *T. gondii* strains were used: type I RH, type II Pru, PYS (ToxoDB #9) strain, and the PruΔ*ku80*Δ*gra47* knockout strain (hereafter referred to as PruΔ*gra47*). Tachyzoites of these strains were propagated in human foreskin fibroblasts (HFFs) cells (SCRC-1041; American Type Culture Collection, Manassas, VA, USA) cultured in DMEM (Gibco, Suzhou, China) supplemented with 2% fetal bovine serum (FBS, Gibco, Melbourne, VIC, Australia) at 37 °C in a 5% CO_2_ incubator. Additionally, Pru cysts were isolated from the brain homogenates of infected mice.

### 2.2. Preparation of Soluble Toxoplasma Antigen (STAg)

Soluble *Toxoplasma* antigen (STAg) was prepared from freshly harvested tachyzoites by repeated freeze–thaw cycles and sonication, followed by centrifugation at 10,000× *g* for 10 min at 4 °C; the supernatant containing STAg was aliquoted and stored at −80 °C until use [23].

### 2.3. Vaccine Potential Assessment of PruΔgra47 Strain

To evaluate the virulence of the PruΔ*gra47* strain, eight-week-old female Kunming mice were intraperitoneally (i.p.) injected with different doses of PruΔ*gra47* tachyzoites (5 × 10^3^, 5 × 10^4^, 5 × 10^5^, 5 × 10^6^, or 5 × 10^7^) or with the wild-type Pru strain (2 × 10^2^ or 5 × 10^4^). Each group contained six mice. Mice were monitored twice daily for clinical signs of toxoplasmosis and mortality for 30 days post-infection. In surviving mice, brain cysts were counted as described in previous studies [16,24].

### 2.4. Detection of Toxoplasma-Specific Antibodies in Immunized Mice

To assess the humoral immune response induced by vaccination, mice received a single i.p. immunization with different doses of PruΔ*gra47* tachyzoites (5 × 10^3^, 5 × 10^4^, 5 × 10^5^, or 5 × 10^6^), and serum samples were collected at 45 days post-immunization. Blood samples were collected via orbital sinus puncture and then placed at 37 °C for one hour. Serum was separated by centrifugation at 4000× *g* for 10 min at 4 °C and stored at −20 °C.

Total IgG, IgG1, and IgG2a antibodies were measured by enzyme-linked immunosorbent assay (ELISA). Microplates were coated with 100 μL/well of STAg (1 μg/100 μL in coating buffer), incubated at 37 °C for 2 h, and then kept at 4 °C overnight. After washing three times with 0.5% PBST, wells were blocked with 5% bovine serum albumin (BSA, VWR Chemicals, Solon, OH, USA) at 37 °C for 2 h. Serum samples were diluted 1:100 in 1% BSA, then 100 μL of the diluted serum was added to each well and incubated at 37 °C for 2 h. HRP-conjugated goat anti-mouse IgG (Abcam, Cambridge, UK) was used at 1:3000 dilution, while goat anti-mouse IgG1 (Abcam, Cambridge, UK) and IgG2a (Abcam, Cambridge, UK) were used at 1:5000 dilution. After three washes, TMB chromogen solution (Beyotime Biotech, Shanghai, China) was added for color development. The reaction was terminated with 2% sulfuric acid, and optical density (OD) was measured at 450 nm. In a single experiment with five biological replicates per group, all ELISAs were performed in triplicate technical replicates for each sample.

### 2.5. Cytokine Detection in Spleen Cell Supernatants

Spleens were harvested from mice that received a single i.p. immunization with PruΔ*gra47* tachyzoites and non-immunized control mice at 45 days post-immunization. After being washed with RPMI-1640 (Solarbio, Beijing, China), the tissues were gently disrupted on a 200-mesh nylon sieve, with gradual addition of RPMI-1640 during the process to minimize cell adhesion. The resulting cell suspension was centrifuged at 1500× *g* for 10 min at 4 °C. The pelleted cells were then exposed to erythrocyte lysis buffer for 3 min to remove red blood cells, followed by thorough washing. Cell viability was assessed using the trypan blue exclusion method, which relies on the principle that viable cells exclude the dye while non-viable cells take it up and appear blue. Only cell preparations with >95% viability were used for subsequent experiments. Viable splenocytes were resuspended in RPMI-1640 supplemented with 10% FBS and adjusted to a density of 3 × 10^6^ cells/mL, then stimulated with 10 μg/mL STAg at 37 °C in a 5% CO_2_ incubator.

Supernatants were harvested at designated time points to measure cytokine levels: 24 h for IL-2 (BioLegend, San Diego, CA, USA) and IL-4 (BioLegend, San Diego, CA, USA); 72 h for IL-10 (BioLegend, San Diego, CA, USA); and 96 h for IL-12 (BioLegend, San Diego, CA, USA) and IFN-γ (BioLegend, San Diego, CA, USA). At each time point, supernatants from three replicate wells were centrifuged at 1500× *g* for 5 min. The clarified supernatants were collected, and cytokine concentrations were determined following the manufacturers’ protocols. In a single experiment, five biological replicates were included per group, and each sample was measured in duplicate technical replicates for all cytokine detection assays.

### 2.6. Protective Effects Against Acute and Chronic Infections

Immunized mice (i.p. inoculated with 5 × 10^6^ PruΔ*gra47* tachyzoites) and control mice (i.p. inoculated with PBS) were challenged at 30 days post-immunization. For acute challenge, mice (*n* = 6 per group) were i.p. infected with tachyzoites of the type I RH strain (10^2^ or 10^3^), PYS strain (10^2^ or 10^3^), or type II Pru (10^6^). Viability and quantity of tachyzoites were confirmed by in vitro plaque assay [16,25]. For chronic challenge, immunized or control mice (*n* = 10 per group) were orally administered 10 or 40 tissue cysts of the Pru strain at 30 days post-immunization. Mortality and clinical symptoms were recorded for 30 days post-challenge. Surviving mice were euthanized, and brain cysts in chronically infected mice were counted microscopically. Brain tissue was further examined by PCR targeting the *B1* gene [26,27].

### 2.7. Statistical Analysis

All experiments were performed once with varying group sizes (*n* = 5 for antibody and cytokine ELISAs, *n* = 6 for virulence and acute protection, and *n* = 10 for chronic protection). Statistical analyses were performed using GraphPad Prism 10.1.2 (GraphPad Software Inc., La Jolla, CA, USA). Normality was first assessed via the Shapiro–Wilk test. The normally distributed antibody data were analyzed by one-way ANOVA followed by Dunnett’s post hoc test, and IgG1/IgG2a subtype comparisons were performed using two-way ANOVA. The non-normally distributed cytokine data were analyzed via the Kruskal–Wallis H test with Dunn’s post hoc test. No statistical analysis was conducted for brain cyst data, which were presented only as scatter plots. The Mantel–Cox log-rank test was applied to assess differences in survival curves, including the calculation of hazard ratios (HRs) and 95% confidence intervals (CIs). A *p* value of <0.05 was considered statistically significant, and *p* values of <0.01, <0.001, and <0.0001 represented increasing levels of significance.

## 3. Results

### 3.1. Virulence Attenuation of PruΔgra47 Knockout Strain in Mice

**Figure 1:**
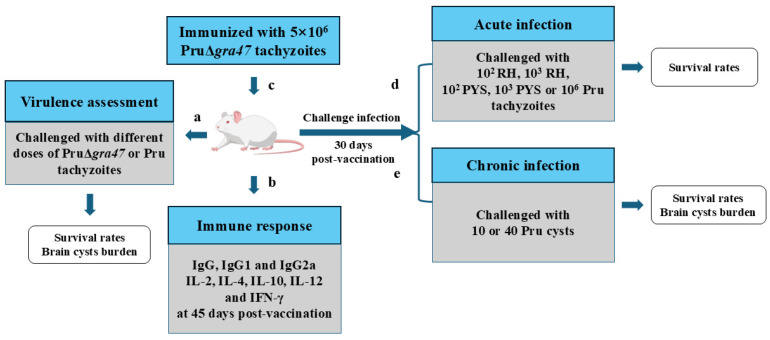
Study design for evaluating the PruΔ*gra47* live-attenuated strain. The schematic includes: (a) virulence assessment; (b) immune response analysis; (c) immunization (5 × 10^6^ tachyzoites); (d) acute challenge with tachyzoites; and (e) chronic challenge with Pru tissue cysts.

To evaluate the virulence and vaccine potential of PruΔ*gra47*, Kunming mice were infected with five different doses of PruΔ*gra47* (5 × 10^3^ to 5 × 10^7^ tachyzoites) or with wild-type Pru (2 × 10^2^ and 5 × 10^4^ tachyzoites). Subsequently, the survival rate of mice in each group and the cerebral cyst burden in surviving mice were analyzed (Figure 1a).

**Figure 2:**
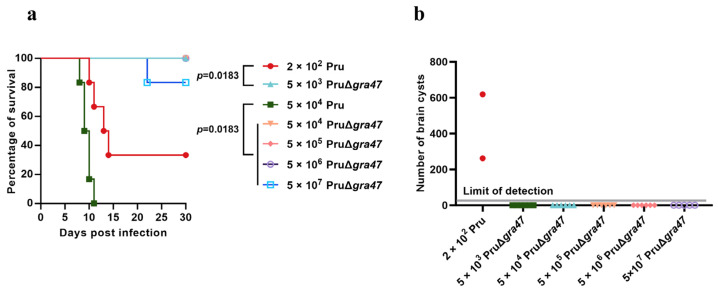
In vivo virulence of the PruΔ*gra47* strain. (**a**) Survival curves of mice after infection with increasing doses of either PruΔ*gra47* or wild-type Pru tachyzoites. Mice were monitored for 30 days post-infection, with 6 mice per group. (**b**) Brain cyst numbers in surviving mice at 30 days post-infection. For the 2 × 10^2^ Pru group, 2 mice were available for cyst counting; for all other groups, 6 mice were in each group.

All mice infected with 5 × 10^4^ tachyzoites of the wild-type Pru strain died within a short period, and only 33% of those infected with 2 × 10^2^ tachyzoites survived. In contrast, all groups of mice infected with PruΔ*gra47* tachyzoites achieved 100% survival except the 5 × 10^7^ group, which still exhibited a survival rate exceeding 80% (Figure 2a).

Compared with mice infected with wild-type Pru tachyzoites, those infected with the PruΔ*gra47* strain showed significantly higher survival rates. Moreover, none of the mice infected with the PruΔ*gra47* strain exhibited obvious clinical symptoms. In contrast, mice in the control group infected with the wild-type Pru strain exhibited clinical symptoms of *Toxoplasma* infection—including lethargy, ruffled fur, and decreased appetite—within one week post-infection, eventually leading to death.

At 30 days post-infection, no brain cysts were detected in PruΔ*gra47*-infected mice, while the two-surviving wild-type-infected mice had brain cyst counts of 619 and 263, respectively (Figure 2b). Collectively, these results confirmed that the virulence of the PruΔ*gra47* strain is markedly attenuated in mice.

### 3.2. Evaluation of the Humoral Immune Response Induced by PruΔgra47 Vaccination

To evaluate the humoral immune response induced by PruΔ*gra47* immunization, serum samples obtained from immunized and nonimmunized mice were used to measure total anti-*Toxoplasma* IgG and IgG subclass levels (Figure 1b).

**Figure 3:**
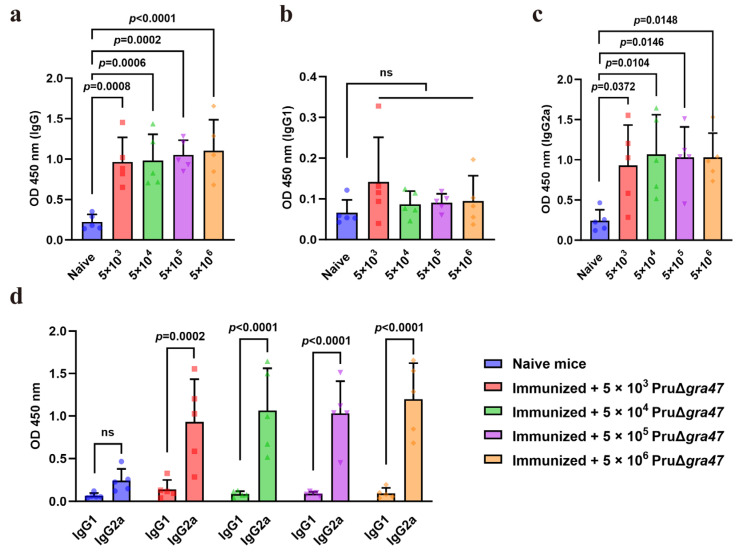
Humoral immune responses at 45 days post-immunization. (**a**–**c**) Serum levels of IgG (**a**), IgG1 (**b**), and IgG2a (**c**) in mice immunized with increasing doses of PruΔ*gra47* tachyzoites. Naive mice served as controls. (**d**) Comparison of IgG1 and IgG2a levels. *n* = 5 mice per group.

Compared with the nonimmunized group, total IgG levels were significantly elevated in all immunized groups and showed a dose-dependent increase, peaking in mice inoculated with 5 × 10^6^ tachyzoites (Figure 3a). IgG subclass analysis revealed that no statistically significant elevation of IgG1 was observed in any immunized group relative to the naive control group (Figure 3b). In contrast, IgG2a levels were significantly elevated in all immunized groups (Figure 3c). However, no significant difference was found between IgG1 and IgG2a levels in the blank control group, whereas IgG2a levels were significantly higher than IgG1 levels in all immunized groups, leading to a predominant IgG2a subclass distribution at these doses (Figure 3d).

Collectively, the sustained increase in total IgG levels indicated that PruΔ*gra47* effectively elicited stable and specific humoral immune responses, laying a foundation for antibody-mediated immune protection. Furthermore, the significant elevation of IgG2a in the absence of a concomitant IgG1 increase suggested that the immunization induced a Th1-skewed immune response.

### 3.3. The Induction of Cell-Mediated Immune Responses by PruΔgra47 Vaccination

To characterize the cell-mediated immune response induced by PruΔ*gra47*, splenocytes were isolated from mice at 45 days post-immunization and stimulated with STAg, after which cytokine secretion levels were measured (Figure 1b).

**Figure 4:**
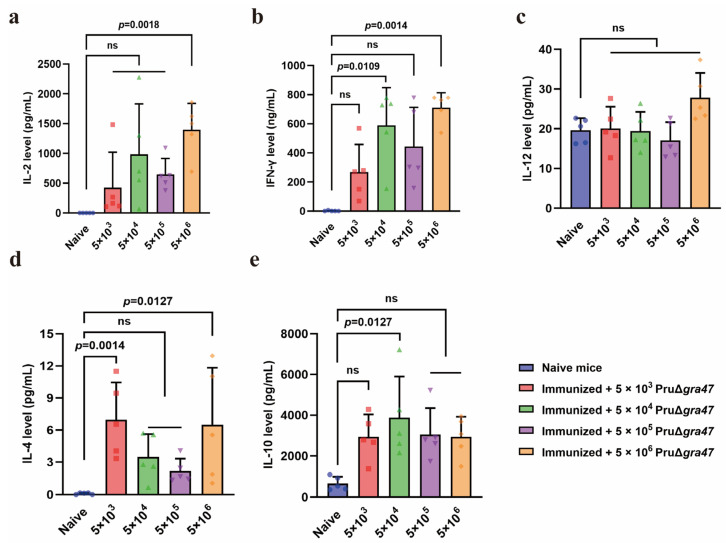
Cytokine responses at 45 days post-immunization. Splenocytes from mice immunized with different doses of PruΔ*gra47* tachyzoites were stimulated with STAg. Levels of IL-2 (**a**), IFN-γ (**b**), IL-12 (**c**), IL-4 (**d**), and IL-10 (**e**) in splenocytes supernatants were measured by ELISA. Naive mice served as controls. *n* = 5 mice per group.

Analysis of cytokine production revealed that immunization with PruΔ*gra47* significantly elevated key Th1-type cytokines in mice. Specifically, IL-2 levels were significantly increased only in the 5 × 10^6^ dose group compared with the naive control group (*p* = 0.0018) (Figure 4a). IFN-γ levels were significantly elevated in both the 5 × 10^4^ (*p* = 0.0109) and 5 × 10^6^ (*p* = 0.0014) immunized groups, with the highest level observed in the 5 × 10^6^ group (Figure 4b). In contrast, IL-12 levels showed no obvious differences in any immunized group relative to the naive control (Figure 4c). Th2-type cytokines displayed a restricted upregulation. IL-4 levels were significantly higher only in the 5 × 10^3^ (*p* = 0.0014) and 5 × 10^6^ (*p* = 0.0127) groups (Figure 4d), whereas IL-10 levels were significantly elevated exclusively in the 5 × 10^4^ group relative to the naive control group (*p* = 0.0127) (Figure 4e).

Taken together, these results demonstrate that PruΔ*gra47* immunization induces a cytokine profile consistent with a Th1-skewed immune response in mice. The 5 × 10^6^ dose group exhibited the strongest Th1-skewed signature, characterized by the highest IFN-γ level and the uniquely significant upregulation of IL-2, a cytokine essential for T-cell proliferation and survival [28]. Protective immunity against *T. gondii* relies primarily on a Th1-type response, in which IFN-γ plays a critical role [29]. Given that the 5 × 10^6^ dose group achieved the most robust IFN-γ response together with significant IL-2 production, this dose was selected as the optimal immunization dose for subsequent experiments.

### 3.4. Immunization with PruΔgra47 Confers Protection Against Acute Infection with T. gondii Tachyzoites

To evaluate the protective efficacy of the PruΔ*gra47* strain against acute *T. gondii* infection induced by different virulent strains of *T. gondii* tachyzoites, challenge experiments were performed on both immunized and naive control mice at 30 days post-vaccination (Figure 1c,d).

**Figure 5:**
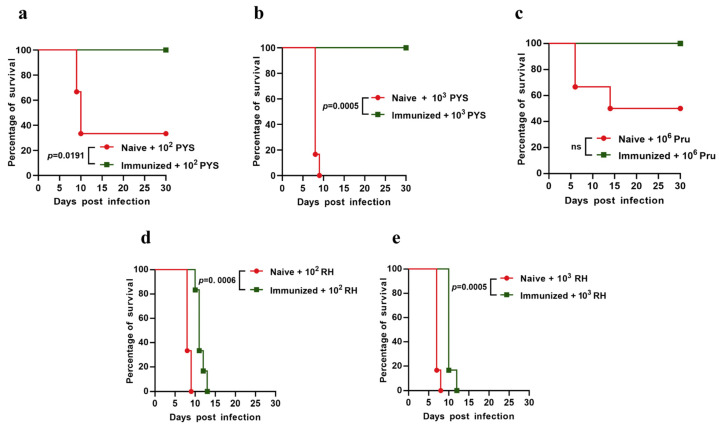
Protection against acute challenge at 30 days post-vaccination. Immunized mice (5 × 10^6^ PruΔ*gra47* tachyzoites) were challenged with different doses of *T. gondii* tachyzoites: (**a**) 10^2^ PYS, (**b**) 10^3^ PYS, (**c**) 10^6^ Pru, (**d**) 10^2^ RH, (**e**) 10^3^ RH. Survival curves are shown for a 30-day observation, with *n* = 6 mice per group.

For the PYS strain (low virulence relative to the RH strain), immunized mice were completely protected (100% survival) against challenge with both doses, whereas naive controls exhibited high mortality, with statistically significant differences at 10^2^ PYS tachyzoites (Figure 5a) and highly significant differences at 10^3^ PYS tachyzoites (Figure 5b). The Mantel-Haenszel hazard ratios for naive mice relative to immunized mice were 12.14 (95% CI: 1.51–97.95) for 10^2^ PYS and 38.76 (95% CI: 5.01–299.8) for 10^3^ PYS (Figure 5a,b). For the homologous Pru strain (10^6^ tachyzoites), immunized mice also showed 100% survival, compared with only 50% survival in naive controls (Figure 5c). The hazard ratio was 10.01 (95% CI: 0.95–105.2); however, the difference in survival did not reach statistical significance for Pru tachyzoite challenge (Figure 5c). For the hypervirulent RH strain, immunized mice displayed significantly prolonged survival by three days, with highly significant differences for both challenge doses (Figure 5d,e): for 10^2^ RH tachyzoites, HR = 28.15 (95% CI: 4.15–190.8); for 10^3^ RH tachyzoites, HR = 38.76 (95% CI: 5.01–299.8) (Figure 5d,e). However, although survival was significantly prolonged, all immunized mice eventually succumbed to the infection in the RH-challenged groups.

Taken together, these results demonstrate that PruΔ*gra47* immunization confers partial protection against acute toxoplasmosis, with complete protection against low-virulence strains (PYS and homologous Pru) and significantly prolonged survival against the hypervirulent RH strain.

### 3.5. Immunization with PruΔgra47 Confers Protection Against Chronic Toxoplasma Infection

**Figure 6:**
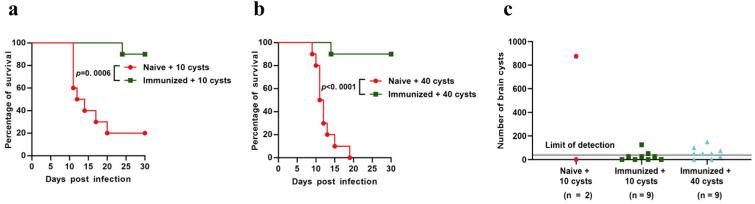
Protection against chronic challenge at 30 days post-vaccination. Immunized (5 × 10^6^ PruΔ*gra47*) and control mice were orally challenged with 10 cysts (**a**) or 40 cysts (**b**) for a 30-day observation, with *n* = 10 mice per group. Survival curves are shown with significance. (**c**) The number of brain cysts in surviving mice at 30 days post-challenge.

To further evaluate the protective potential of PruΔ*gra47* against chronic *T. gondii* infection, immunized and naive mice were orally challenged with 10 or 40 cysts of the Pru strain at 30 days post-vaccination (Figure 1e). Immunized mice exhibited a significantly higher survival rate (maintained at 90%) without obvious clinical signs of toxoplasmosis, whereas control mice showed marked susceptibility. Among control mice infected with 10 cysts, deaths began on day 11, resulting in a final survival rate of only 20%, which was highly significantly different from the immunized group (*p* = 0.0006) (Figure 6a). All control mice infected with 40 cysts died within 20 days, showing an extremely significant difference in survival rate compared with the immunized group (*p* < 0.0001) (Figure 6b). The Mantel-Haenszel hazard ratios for naive mice relative to immunized mice were 12.59 (95% CI: 2.95–53.86) for the 10-cyst challenge and 19.94 (95% CI: 5.19–76.61) for the 40-cyst challenge (Figure 6a,b).

Collectively, these data demonstrate that immunization with PruΔ*gra47* markedly improves survival following oral challenge with Pru tissue cysts and significantly reduces the risk of death during chronic *T. gondii* infection.

### 3.6. Vaccination with the PruΔgra47 Strain Reduced Brain Cyst Burden in Chronically Infected Mice

To determine the effect of vaccination on brain cyst burden during chronic *T. gondii* infection, brain tissues were collected from mice that survived for 30 days post-challenge for cyst counting. In the unimmunized group, the two mice that survived challenge with 10 cysts had brain cyst counts of 875 and 0, respectively (Figure 6c). In contrast, the median cyst counts in PruΔ*gra47*-immunized animals were 25 (10-cyst challenge) and 50 (40-cyst challenge) (Figure 6c). The brains of all surviving mice were examined by PCR targeting the *T. gondii B1* gene. The results revealed that, among immunized mice surviving challenge, the *B1*-positive rate was 77.8% for both cyst doses, with some mice testing PCR-positive despite having no visible cysts. These results indicate that, despite a reduction in brain cyst burden, *T. gondii* DNA persists in the majority of immunized mice and a proportion still harbor detectable cysts, suggesting the remaining risk of infection reactivation.

## 4. Discussion

As key secretory proteins within *T. gondii*, GRAs are widely recognized as important candidate proteins for the development of *Toxoplasma* vaccines. GRA72 interacts with GRA47 to possibly form heteromeric pore channels on the PVM, regulating PVM permeability and the localization of GRA17 and GRA23 on the PVM [21]. It has been established that both GRA17 and GRA23 are significant virulence factors of *T. gondii*. These gene-deficient strains are vaccine candidates for live-attenuated toxoplasmosis vaccines [30,31]. GRA47 is functionally homologous to GRA17 and GRA23, and this study systematically evaluated the safety, immunogenicity, and in vivo immunoprotective efficacy of PruΔ*gra47* as a live-attenuated vaccine candidate. Virulence assays revealed that the PruΔ*gra47* strain exhibits substantially attenuated virulence. This observation was made evident by the intraperitoneal injection of 5 × 10^6^ of PruΔ*gra47* tachyzoites to mice, which resulted in a 100% survival rate without manifesting overt clinical symptoms. Furthermore, the formation of cerebral cysts was found to be reduced. These findings suggest the potential of the PruΔ*gra47* strain as a candidate vaccine for toxoplasmosis.

Immunization with PruΔ*gra47* elicited a Th1-skewed immune response, which is important for protection against *T. gondii* [32,33,34,35]. This was evidenced by the marked elevation of IgG2a in the immunized mice, whereas IgG1 levels remained comparable to naive controls, a typical feature of Th1-biased humoral immunity associated with enhanced resistance to intracellular pathogens [36,37]. Furthermore, immunization significantly increased Th1-type cytokines, notably IFN-γ and IL-2 at the highest immunization dose. These cytokines play central roles in mediating cellular immunity and inhibiting *Toxoplasma* proliferation in host cells [38]. The IFN-γ-dominant response contributed to effective protection against both acute and chronic toxoplasmosis [39]. In addition, selective elevation of IL-4 and IL-10 was observed at certain doses, which may suppress IFN-γ secretion and mitigate Th1-driven tissue damage [40]. Of note, cytokine data alone only suggest Th1 skewing rather than confirming absolute Th1 polarization, future T-cell subset phenotyping is required to precisely verify immune polarization status.

Notably, no significant elevation of IL-12 was observed at any immunization dose. The lack of IL-12 increase may be attributed to the late analysis time point (45 days post-immunization) or immune evasion mechanisms of *T. gondii* [41]. The moderate upregulation of IL-4 and IL-10 likely exerts an immunomodulatory effect, preventing excessive inflammation caused by an over-activated Th1 response [42,43]. This Th1-skewed profile is considered beneficial for controlling parasite burden and limiting immunopathology, representing an ideal immune profile for an attenuated live vaccine [24,44]. Collectively, these findings confirm that PruΔ*gra47* induces a protective and well-balanced immune response, demonstrating potential as a relatively safe and effective attenuated live vaccine candidate.

Acute *T. gondii* infection can lead to severe pathological consequences including fatal encephalitis, especially when caused by the highly virulent Type I RH strain. Meanwhile, the ToxoDB#9 genotype (e.g., the PYS strain) that is highly prevalent in China also represents a considerable threat to public health. In the present study, immunization with PruΔ*gra47* provided complete protection against challenge with the Type II Pru strain and the prevalent Chinese ToxoDB#9 strain. Although complete protection was not achieved against the hypervirulent Type I RH strain, the attenuated PruΔ*gra47* strain still significantly prolonged the survival time of infected mice. The relatively lower protective efficacy against the RH strain can be attributed to multiple factors. First, Kunming mice used in this study are inherently more susceptible to toxoplasmosis with higher mortality than BALB/c mice [17]. Second, the Type I RH strain is extremely virulent and can kill mice with even a single tachyzoite, which may far exceed the clearance capacity of most vaccine-induced immune responses. Third, the attenuated PruΔ*gra47* strain was derived from the Type II Pru strain rather than Type I RH, so the induced immune response may not fully cover the antigenic spectrum of the heterologous RH strain. These differences in host susceptibility, pathogen virulence, and antigenic background may collectively explain the limited protection against the Type I RH strain observed in this study [17].

Chronic infection is a major pathological hazard of toxoplasmosis, as *T. gondii* can form latent cysts in the brain and other tissues which may reactivate upon host immunosuppression, leading to recurrent and life-threatening clinical manifestations. In the present study, we mimicked natural infection via oral challenge with tissue cysts, and found that mice immunized with PruΔ*gra47* exhibited a 90% survival rate, with markedly fewer brain cysts than the surviving control mice. Given the limited number of surviving control animals, formal statistical comparison was not performed. These low cyst observations suggest that PruΔ*gra47* may inhibit cyst formation and potentially lowering the risk of reactivation during chronic toxoplasmosis. The protective efficacy against chronic infection may be attributed to the predominantly Th1-skewed immune response induced by vaccination, which are known to restrict cyst development [32,33]. Notably, the presence of PCR-positive/cyst-negative mice indicates that PruΔ*gra47* vaccination does not provide sterilizing immunity. However, the reduction in visible brain cyst burden has significant clinical relevance, as cyst load correlated with reactivation risk and disease severity in immunocompromised individuals. Even if microscopic cysts persist, the reduction in cyst load would substantially lower the risk of clinical toxoplasmosis. Therefore, as a live-attenuated vaccine candidate, PruΔ*gra47* confers effective protection against both low-virulence *Toxoplasma* strains and cyst-mediated chronic infection.

Several live-attenuated *T. gondii* strains have been reported, including Δ*gra72*, Δ*cdpk3*, Δ*pp2a-c*, Δ*gra17* and Δ*gra23* mutants. In terms of safety compared with these mutants based on published data, PruΔ*gra47* exhibited 100% survival at 5 × 10^6^ tachyzoites and 83.3% survival at the dose of 5 × 10^7^ tachyzoites, with no detectable brain cysts, a “cyst-free” phenotype comparable to PruΔ*gra72* and PruΔ*pp2a-c* [16,17], whereas PruΔ*gra23* retained residual cyst-forming ability [31]. Regarding the efficacy against low-virulence strains, PruΔ*gra47* provided 100% protection against both the homologous type II Pru strain (10^6^ tachyzoites) and the heterologous Chinese epidemic ToxoDB#9 PYS strain (10^2^ or 10^3^ tachyzoites), comparable to PruΔ*gra72* [16]. Against the hypervirulent type I RH strain, PruΔ*gra47* significantly prolonged survival but did not achieve complete protection, similar to PruΔ*pp2a-c*, and reflecting the extreme virulence of the RH strain and Type I/II antigenic disparity [17]. Following oral challenge with Pru tissue cysts, PruΔ*gra47* immunization achieved 90% survival and a reduction in brain cyst burden, comparable to PruΔ*gra72* and PruΔ*pp2a-c* [16,17]. No vaccine candidate achieves sterilizing immunity; however, the reduction in brain cyst burden is clinically valuable for lowering the reactivation risk in immunocompromised individuals. Although PruΔ*gra47* did not outperform these live-attenuated *T. gondii* strains, this study offers a valuable experimental basis and a new direction for future development of live-attenuated vaccines against toxoplasmosis.

Several limitations of the present study should be acknowledged. First, the vaccination efficacy of PruΔ*gra47* was only evaluated in a mouse model, with no validation in food-producing animals. Second, challenge experiments were conducted only at 30 days post-immunization; thus, only short-term immunoprotective effects were assessed, and the long-term persistence of immunity remains to be determined. Third, the induction and maintenance of immune memory were not fully investigated. In addition, protection against congenital infection and oocyst-mediated infection—both important natural transmission routes—was not evaluated. Furthermore, the safety assessment was incomplete, key safety studies were not performed, such as histopathology, systemic parasite dissemination, virulence reversion, and clinical/behavioral monitoring, limiting the comprehensiveness of our safety conclusion. Future studies should address these limitations in large animal models and explore long-term protection and immune memory.

## 5. Conclusions

In summary, the present study demonstrates that the PruΔ*gra47* mutant induces a predominantly Th1-skewed immune response against *T. gondii* infection. Immunization with PruΔ*gra47* provides effective protection against challenge with low-virulence tachyzoites and cysts, moderately prolongs survival against highly virulent challenge, and leads to a reduction in brain cyst burden during chronic infection. Although the protective efficacy is not optimal, these findings suggest that PruΔ*gra47* may still hold potential as a live-attenuated vaccine candidate and warrants further evaluation in food-producing animals and cats against toxoplasmosis.

## Data Availability

All data generated or analyzed during this study are included in this published article and its Supplementary Materials. For additional information, please contact the corresponding authors.

## Supplementary Materials

The following supporting information can be downloaded at: https://www.mdpi.com/article/10.3390/ani16131964/s1. Figure S1: PCR detection of *T. gondii B1* gene in brain tissues from immunized mice challenged with 10 or 40 Pru cysts, A1–9: results from the surviving mice challenged with 10 cysts; B1–9: results from the surviving mice challenged with 40 cysts. P: positive control; N: negative control; Table S1: Brain cyst burden and *B1* gene detection results of the mice challenged with Pru cysts.
