# Supplementary material for: The Live-Attenuated PruΔgra47 Strain of Toxoplasma gondii Confers Protective Immunity Against Acute and Chronic Toxoplasmosis in Mice
Source: Animals (Basel). 2026 Jun 25;16(13):1964. doi: 10.3390/ani16131964 (PMC13360131; doi:10.3390/ani16131964)
Supplement: Supplementary file 1 [file animals-16-01964-s001.zip › Table S1.pdf]

Table S1. Brain cyst burden and *B1* gene detection results of the mice challenged with Pru cyst

Group	Average brain cyst number	Positive rate of *B1* gene	Brain cyst number (*B1* gene detection result)								
			1	2	3	4	5	6	7	8	9
Naive +10 cysts	437.5	50%	875	0	-	-	-	-	-	-	-
Immunized +10 cysts	27.8	77.8%	0 (-)	0 (-)	25 (+)	25 (+)	0 (+)	50 (+)	125 (+)	25 (+)	0 (+)
Immunized + 40 cysts	55.6	77.8%	0 (-)	0 (-)	50 (+)	150(+)	50 (+)	25 (+)	50 (+)	100 (+)	75 (+)
