# Supplementary figures and images for: The Live-Attenuated PruΔgra47 Strain of Toxoplasma gondii Confers Protective Immunity Against Acute and Chronic Toxoplasmosis in Mice
Source: Animals (Basel). 2026 Jun 25;16(13):1964. doi: 10.3390/ani16131964 (PMC13360131; doi:10.3390/ani16131964)

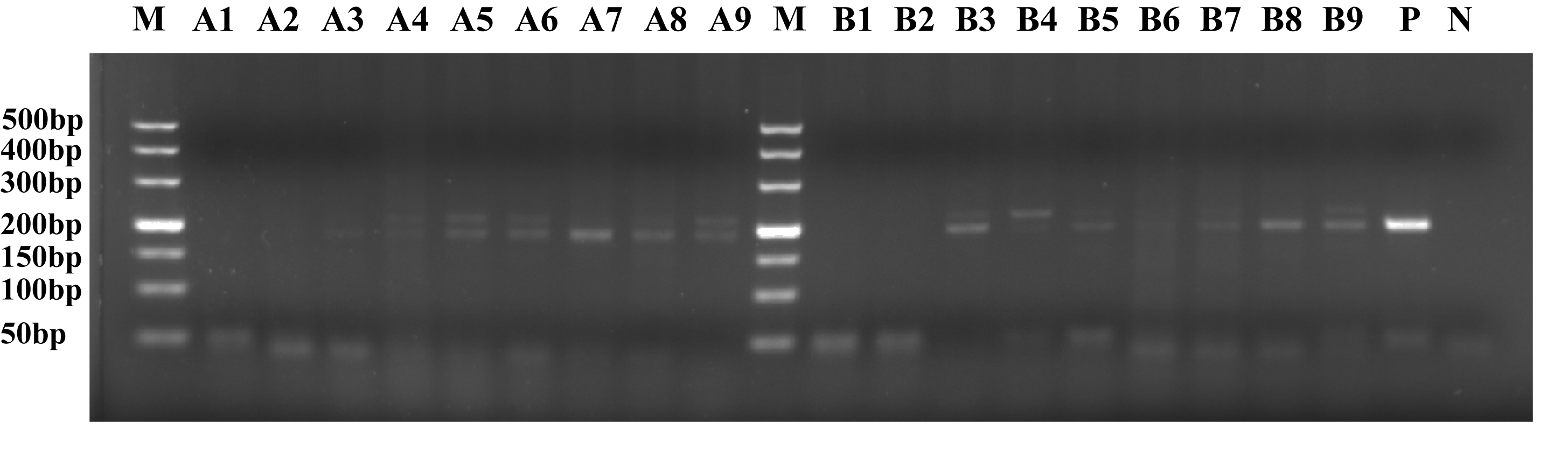

Supplement: Supplementary file 1 [file animals-16-01964-s001.zip › Figure S1.tif]
